# Hypernetworks Reveal Compound Variables That Capture Cooperative and Competitive Interactions in a Soccer Match

**DOI:** 10.3389/fpsyg.2017.01379

**Published:** 2017-08-28

**Authors:** João Ramos, Rui J. Lopes, Pedro Marques, Duarte Araújo

**Affiliations:** ^1^ISCTE-Instituto Universitário de Lisboa Lisbon, Portugal; ^2^Universidade Europeia, Laureate International Universities Lisboa, Portugal; ^3^Instituto de Telecomunicações Lisbon, Portugal; ^4^Football Performance, City Football Services Manchester, United Kingdom; ^5^Interdisciplinary Centre for the Study of Human Performance (CIPER), Faculdade de Motricidade Humana, Universidade de Lisboa Lisbon, Portugal

**Keywords:** network theory, hypernetworks, network dynamics, performance analysis, soccer

## Abstract

The combination of sports sciences theorization and social networks analysis (SNA) has offered useful new insights for addressing team behavior. However, SNA typically represents the dynamics of team behavior during a match in dyadic interactions and in a single cumulative snapshot. This study aims to overcome these limitations by using hypernetworks to describe illustrative cases of team behavior dynamics at various other levels of analyses. Hypernetworks simultaneously access cooperative and competitive interactions between teammates and opponents across space and time during a match. Moreover, hypernetworks are not limited to dyadic relations, which are typically represented by edges in other types of networks. In a hypernetwork, *n-ary* relations (with *n* > 2) and their properties are represented with hyperedges connecting more than two players simultaneously (the so-called *simplex*—plural, *simplices*). Simplices can capture the interactions of sets of players that may include an arbitrary number of teammates and opponents. In this qualitative study, we first used the mathematical formalisms of hypernetworks to represent a multilevel team behavior dynamics, including micro (interactions between players), meso (dynamics of a given critical event, e.g., an attack interaction), and macro (interactions between sets of players) levels. Second, we investigated different features that could potentially explain the occurrence of critical events, such as, aggregation or disaggregation of simplices relative to goal proximity. Finally, we applied hypernetworks analysis to soccer games from the English premier league (season 2010–2011) by using two-dimensional player displacement coordinates obtained with a multiple-camera match analysis system provided by STATS (formerly Prozone). Our results show that (i) at micro level the most frequently occurring simplices configuration is 1vs.1 (one attacker vs. one defender); (ii) at meso level, the dynamics of simplices transformations near the goal depends on significant changes in the players' speed and direction; (iii) at macro level, simplices are connected to one another, forming “simplices of simplices” including the goalkeeper and the goal. These results validate qualitatively that hypernetworks and related compound variables can capture and be used in the analysis of the cooperative and competitive interactions between players and sets of players in soccer matches.

## Introduction

Coaches, players, and scientists have long tried to understand team behavior dynamics during a game, aiming to develop interventions and training plans that may increase team performance (Araújo and Davids, 2016; Passos et al., 2017). Broadly speaking, research in performance analysis in team sports searches for variables describing game dynamics that are: (i) useful and accessible to coaches and athletes; (ii) obtained automatically or semi-automatically from game observation; and (iii) related to team outputs, such as, match results. For finding such variables it is necessary to capture the multi-leveled dynamics emerging from differential interactions between many heterogeneous parts (e.g., players), while considering potential adaptations to changing environments. In this way, teams and athletes can be seen as co-evolving subsystems that self-organize into new structures and behaviors (Johnson, 2013), i.e., they form team synergies (Araújo and Davids, 2016). Such team synergies emerge from physical and informational constraints (Schmidt et al., 1998, 2011). Importantly players are perceptually linked mainly by informational constraints, since physical links among them are very rare (e.g., when forming a wall of players; Riley et al., 2011). Several studies have analyzed the coupling among performers based on interpersonal distance measures (Passos et al., 2011; Fonseca et al., 2013; Rio et al., 2014), with a higher emphasis on the distance between a player and the immediate opponent (e.g., Headrick et al., 2012). In the present study, we extend this player-immediate opponent distance to the closest player (opponent or not).

These interactions, based on informational and physical constraints have been studied by network theorical approaches, like social network analysis (SNA). SNA is a powerful tool to capture and study interpersonal relations in team sports (Araújo and Davids, 2016); however, this method can only be used for representing binary (*2-ary*) relations (Johnson, 2006; Criado et al., 2010; Boccaletti et al., 2014). The most common graphical representations of SNA depict players as nodes in fixed positions in the pitch (the field of the match), with edges between them representing the cumulative “ball flux,” i.e., ball passes, over time (Duch et al., 2010; Fewell et al., 2012; Grund, 2012; Clemente et al., 2015; Araújo and Davids, 2016; Travassos et al., 2016). This is a fundamental limitation of typical SNA in sport context, as it restricts its application to the attacking phase of team dynamics. Typically, all other relevant types of interactions, either cooperative or competitive, are not considered. In this study, we investigate how cooperative (e.g., between players of the same team in order to create a scoring opportunity) and competitive interactions (e.g., between players of different teams competing for ball possession) may be captured and analyzed via multilevel hypernetworks. On the one hand, according to Boccaletti et al. (2014), multilevel networks constitutes the new frontier in many areas of science since it describes systems that are interconnected through different categories of connections (e.g. relationship: teammate vs. opponent; activity: increasing vs. diminishing interpersonal distance; category: attacker vs. midfielder) that can be represented in multiple layers, including networks of networks (e.g., interactions between teams). On the other hand, in a hypernetwork, a hyperedge can connect more than two nodes, thus directly representing *n-ary* interactions occurring among small sets of nodes, 〈*p*_*i*_, …, *p*_*j*_〉 (Johnson, 2006, 2008, 2013, 2016; Criado et al., 2010; Boccaletti et al., 2014). This generalization provided by hypernetworks enables the representation of cooperative and competitive interactions that occur during the game and that involve an arbitrary number of players (teammates or opponents).

In the present study, we have extended the approach by Johnson and Iravani (2007) by introducing compound variables, e.g., local dominance, which capture the structure and dynamics of cooperative and competitive interactions in the following ways:

- By considering the domain specificity of soccer matches to tag the sets of players formed (e.g., 2 vs. 1 corresponds to a set with two attackers and one defender) as these tags describe local dominance (Duarte et al., 2012);
- By including the spatiotemporal occurrence of the different sets of players by counting their frequency and location;
- By analyzing and relating the dynamics of the sets with players velocity in specific events (goal scoring opportunities);
- By studying, for the same events of interest, the formation and dynamics of higher level simplices; notably, the relations between simplices of simplices.

The present approach is applied to a set of matches in order to investigate how the proposed compound variables can be useful on characterizing the behavior of players and teams at different levels and the relationships between these levels and match context, e.g., team local dominance and current match result.

As a first step in this approach, it is necessary, at each level of analysis, to identify the meaningful relations for the match dynamics, and represent them using different criteria for selecting the players in each set (i.e., connected by a hyperedge; Johnson, 2008, 2016). According to Passos and colleagues the analysis of the interpersonal distances is adequate for complex systems modeling (Passos et al., 2011). As we are interested in cooperative and competitive behavior in the pitch, geographical proximity between players (Headrick et al., 2012) can capture whether an interaction between players exists or not (e.g., functional couplings). Also, in the investigation of the relation between higher (macro) level of analysis and players' individual actions (micro), it is important to consider the velocity of each player, as well as the velocity of the set of players, represented by the set's geometric center and obtained through the computation of each players' velocity. For example if such set is expected to maintain its structure or if it is about to split when a player's velocity vector is moving away from the other players. Operationally, we have defined that a player does interact with his closest player; this interaction is cooperative when that closest player is a teammate, and competitive when it is an opponent. Thus, time and space are highlighted in the present approach using hypernetworks because it uses geographical proximity criteria, and also because it captures temporal changes, by considering the players' geographical positions over time (*t*_1_, *t*_2, …_, *t*_*n*_). The compound variables adopted in this study reflect and capture this space and temporal features, e.g., local dominance and the dynamics, i.e., changes on, players' sets.

**Figure 1:**
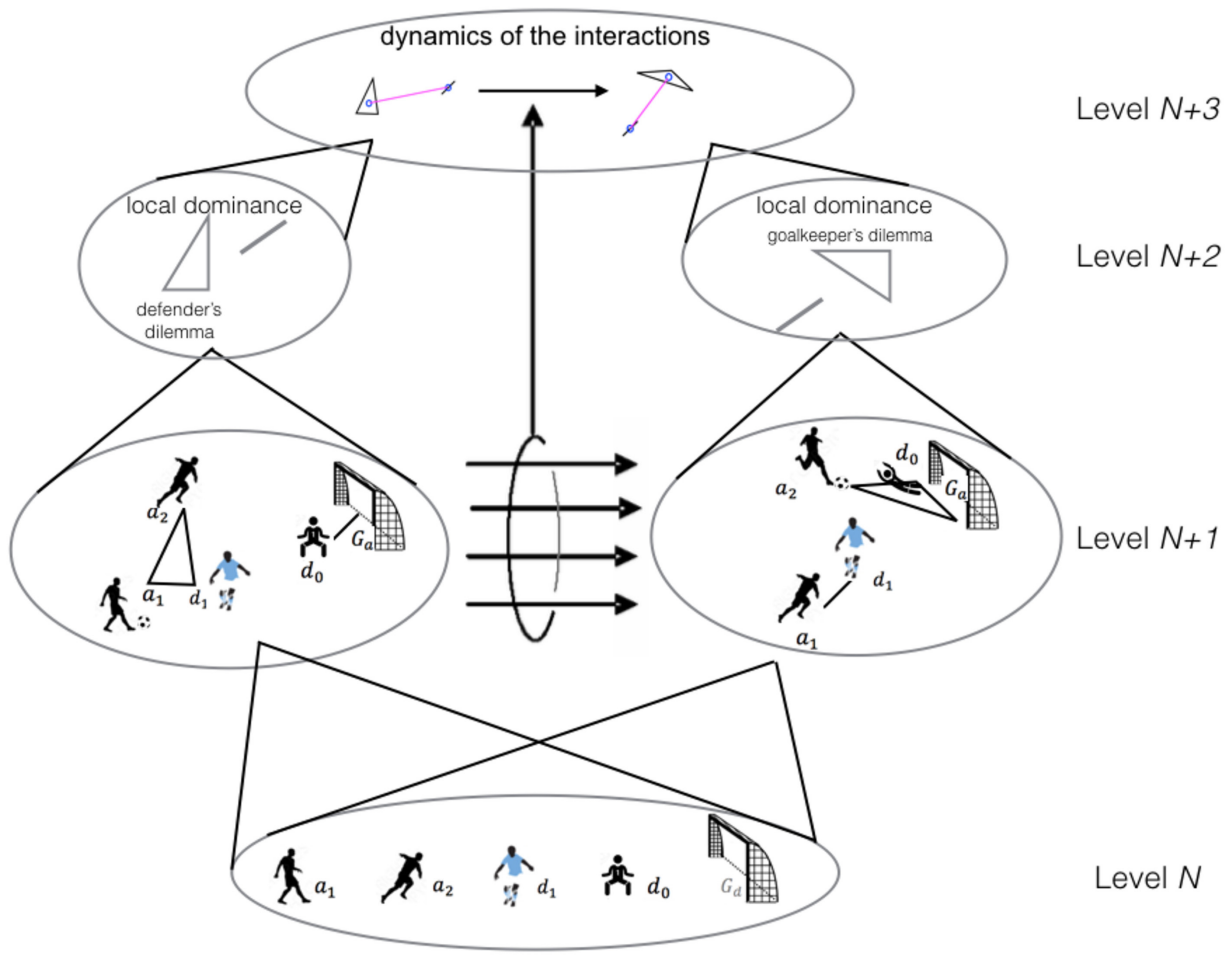
Multilevel hypernetwork representation (from bottom to top). Each level corresponds to a different abstraction level (Level *N*, players in the pitch; Level *N* + 1, proximity-based simplices; Level *N* + 2, local dominance relation; Level *N* + 3, dynamic analysis via simplices of simplices). Also represented, the displacement in a soccer game of 2 sequential time frames (from the left to the right hand side) (Adapted from Johnson and Iravani, 2007).

In Figure 1, we show an example of a set of nodes identified at Level *N*: two attacking players (*a*_1_ and *a*_2_,), a defender (*d*_1_), a goalkeeper (*d*_0_), and a goal (*G*_*a*_). These nodes are connected by two hyperedges at Level *N* + *1*, corresponding to sets 〈*a*_1_, *a*_2_, *d*_1_〉 and 〈*d*_0_, *G*_*a*_〉 in one time frame, and 〈*a*_1_, *d*_1_〉 and 〈*a*_2_, *d*_0_, *G*_*a*_〉 on the next.

For a more complete description of the system's dynamics, each tuple identified in the hypernetwork can be extended by an element, *R*, that describes the relationships in the set (Johnson, 2013). Each of these extended sets is called a *simplex* (Johnson and Iravani, 2007; Johnson, 2013). For example, *R* is the path to understand why the sets 〈*a*_1_, *a*_2_, *d*_1_〉 and 〈*d*_0_, *G*_*a*_〉 on one frame lead to the sets and 〈*a*_1_, *d*_1_〉 and 〈*a*_2_, *d*_0_, *G*_*a*_〉 on the next. When a player observes the game searching for the best action possibilities offered by the other players' positioning, the entire configuration of team-mates and opponents has to be perceived. Such sets of players, either in 1vs.1, 2vs.1, or 2vs.2, or any other set, may be related to one another, regarding the players' general configuration. Thus, when one player decides to move, the entire configuration is affected. Johnson and Iravani (2007) propose naming the “2 attackers vs. 1 defender” structure, the *defenders' dilemma*, since the defenders can opt to tackle the ball or intercept the pass between attackers. In a similar situation involving the goalkeeper, the *goalkeepers' dilemma*, the options are moving to the right or left of the goal, or moving toward the attacker leaving the goal behind. The goal can therefore be considered as a constraint that attracts the opponents and instigates the defenders to position as if it were an opponent. For this reason, we have included goals in the definition of simplices, because they show similarities to an “attacking player” (e.g., in the goalkeepers dilemma).

In this study, we propose several compound variables to describe the players' cooperative and competitive behavior dynamics during a soccer match. The simplest of these variables depicts the dominant interactions in each set, and is expressed by two values representing the number of attacking and defending players, for example, 2 vs. 1 corresponds to a set with two attackers and one defender. In Figure 1, the two dominant relationships are *R*_1_ = (2 *vs*.1) and *R*_2_ = (0 *vs*.1), and the corresponding simplices are σ_1_ = 〈*a*_1_, *a*_2_, *d*_1_; (2 *vs*.1)〉 and σ_2_ = 〈*d*_0_, *G*_*a*_; (0 *vs*.1)〉. The behavior of a team during a match can then be described by other compound variables that characterize the relative frequencies of the aforementioned relationships. For example, the minimal structure (simplex) of players' interactions occurring more frequently in a match can be assessed.

At higher complexity levels, the hypernetwork can represent the interactions between related simplices, or simplices of simplices (see Figure 1, *Level N* + *3;* Johnson, 2006, 2013; Johnson and Iravani, 2007). In what regards the study of dynamics: less dynamic structures (e.g., number of players, players' roles, etc.) are called *backcloth*, and higher rate changes (e.g., players positioning in relation to opponents, teammates and the goal or the ball) are called *traffic* (Johnson, 2013) and represent dynamics within the backcloth. Thus, one important feature of hypernetwork analysis in the sports context is the representation of players' *moves*, across time and space, and between structured sets (i.e., from one simplex to another). As shown in Figure 1, this multilevel approach allowed us to capture the number of players and their moves and the players in the match-day squad (*Level N*), the coordinated sets of players along the match (*Level N* + *1*), the local advantage of one team over the other (e.g., numerical dominance; *Level N* + *2*), and the relationship between the sets (*Level N* + *3*). Moreover, by using this approach different compound variables, e.g., local dominance, may explain distinctive aspects of the competitive and cooperative behavior of players and teams.

In this study we put forward the hypothesis that hypernetworks and compound variables over these hypernetworks can capture relevant features of soccer team dynamics during a match. We validate qualitatively this hypothesis by applying the proposed method to a set of matches of a focal team within different contexts and by analysis the results thus obtained. The aim of this study was therefore to operationalize a method addressing different levels of hypernetworks on soccer matches and by providing a study case for tackling the following questions:

- At *Level N*: Has the backcloth (players) changed during the match, as expressed by events such as, substitutions, sent-offs and injuries? Typical notational analyses answer this question directly.
- At *Level N* + *1*: What are the most frequently occurring simplices in soccer matches? A histogram with the relative frequencies of occurrence of every type of simplices (e.g., 1vs.1, 2vs.1…) can be computed.
- At *Level N* + *1*: Are there any differences in simplices' structure and occurrence between home or away matches for Team A? A heat map (2D spatial frequency map) for each of the relationships can be computed to show their location in the pitch.
- At *Level N* + *1*: Are there any changes in simplices structure and field position as the match score changes? Instead of considering the entire match, the heat maps can address specific periods of the match. These periods are bounded by relevant match events, e.g., a goal being scored.
- At *Level N* + *2*: What are the dynamics of the simplices' interactions near the goal, immediately before the score changed? Instead of examining the results for the entire match, or for given periods, it is possible to perform a frame-by-frame analysis to assess which simplices formed and how they changed, and also to identify the players who contributed to those changes.
- At *Level N* + *3*: Is there any interaction between simplices leading to the emergence of new team configurations that, in turn, can lead to scoring a goal? To answer this question, it is necessary to evaluate how the different simplices relate to one another, how they aggregate into higher-level simplices, and how they recombine into different simplices.

## Methods and materials

Five matches were analyzed from a pool of 11 matches of the English Premier League season 2010–2011 provided by STATS (formally Prozone). This data set was selected because it contained no errors, such as, missing or duplicated positioning data, and because the *backcloths* were equivalent (i.e., there were no differences between teams regarding the number of players due to sent-offs or injuries without substitutions). Participants included all the players in the field from Team A (our focal team), and the players from five teams playing against team A (teams B, C, D, E, and F). The matches included three home matches, against teams B, C, and D, and two away matches, against teams E and F. The players' substitutions were considered but not analyzed in detail in this study (i.e., data for both initial squad and substitutes are used but the implications of substitutions in the backcloth are not taken into consideration).

Matches and their score were: Team A vs. Team B (1–0); Team A vs. Team C (1–0); Team A vs. Team D (1–0); Team E vs. Team A (2–1) and Team F vs. Team A (0–0). The details for each match are presented in Table 1.

**Table 1:** Matches' details indicating the result and changes in the team structure due to sent-offs, substitutions, or injuries (without substitution).

**Matches**	**A vs. B**	**A vs. C**	**A vs. D**	**E vs. A**	**F vs. A**
Results	1–0	1–0	1–0	2–1	0–0
Substitutions	3–3	3–3	3–3	3–3	2–2
Sent-offs	0–0	0–0	0–0	0–0	1–1
Injuries (without substitution)	0–0	0–0	0–0	0–0	0–0

**Figure A1:**
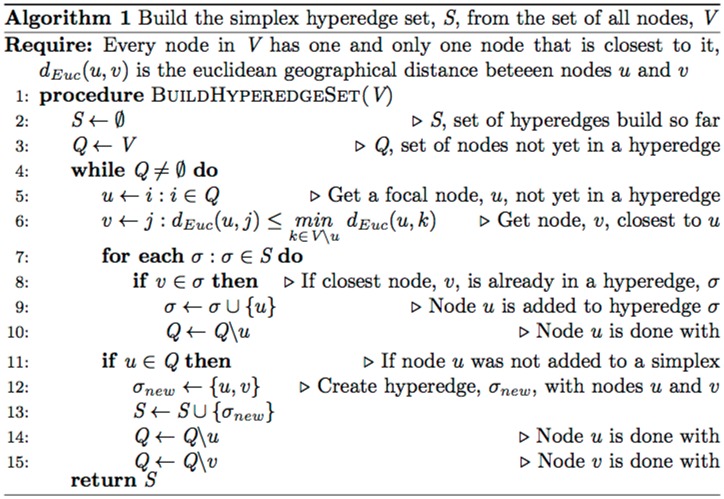
Pseudocode for building the simplex hyperedge set.

For each match, raw data consisted of two-dimensional player displacement coordinates provided by STATS. These data were obtained by a multiple-camera match analysis system whereby the movements of the 22 players during the match were recorded with eight cameras positioned at the top of the stadium. The frames were processed at 10 Hz through an automated system that synchronized the video files. The effective playing area was 80 m wide and 120 m long, including the out-of-bound locations such as, set-plays. A computer procedure for computing the simplices' hyperedges set with the proximity criterion was implemented using GNU Octave version 4.2.0 and applied to each frame. This criterion has the advantage of being non-parametric; the corresponding pseudo-code for this algorithm is provided in Figure A1.

Each simplex was represented graphically by the convex hull computation (the minimum convex area containing all players in the simplex) and included the velocity of each player (vector velocity considering the instant *t-1* and *t*), as well as the velocity of the geometric center of the simplices.

To represent the field positioning of the different types of simplices, we used heat maps for the frequency of simplices occurrence. This type of graphical representation allowed us to capture the most frequent type of simplices for each time period, as well as their geographical position in the field.

For analyzing specific time points, we represented simplices (*Level N* + *2*, **Figures 5**, **6**) with two different colors: for players in team A, vertices are in **red**, for players in team B, vertices are in **green**. For the higher-level simplices in level *N* + *3*, **Figure 6**, the blue **o** symbol represents the geometric center of the simplices. Such representation facilitates the simultaneous identification of players in both teams and the type of simplices in level *N* + *3*. Moreover, we also represented the proportion (local dominance or balance) of each type of simplices in level *N* + *2*, as well as the type of relation that exists between the simplices, or simplices of simplices in any instant of time at level *N* + *3*. The velocity of the simplices and players were also included, thus allowing for the evaluation of simplices consistency, for example, transformations such as, when a player entered or moved away from a given simplice, or when all players moved simultaneously to the same position, could be detected.

## Results

Our results revealed how the matches' hypernetworks are characterized from *Level N* to *Level N* + *3*.

We analyzed the structure at *Level N* of the five matches. As expected, we found 11 players in each team, with some players being substituted but with no sent-offs (with the exception of match F vs. A) or injuries occurring after there were no substitutions left (hence the total number of players remained constant). At this level of analysis, individual player statistics and heat maps of their positioning during the match are usually performed. However, as this type of performance analysis is widespread in sport (for a review see Passos et al., 2017), and given that the focus of this paper is on team behavior, we do not present such results here.

**Figure 2:**
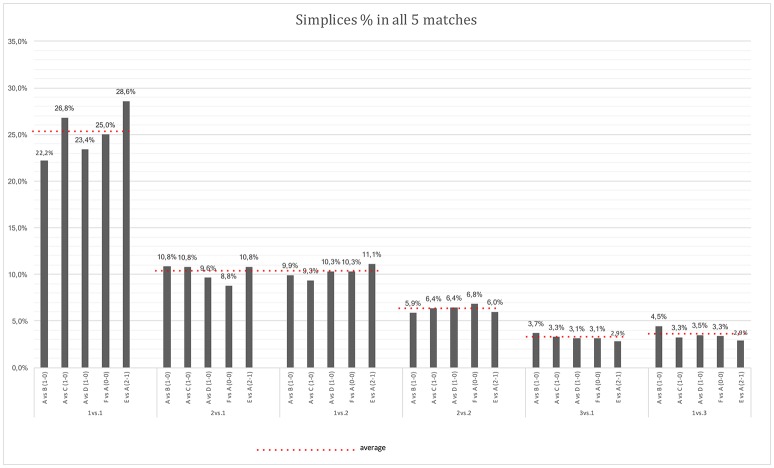
Histogram for the most frequently occurring simplices structures in the 5 matches: 1vs.1; 2vs.1; 1vs.2; 2vs.2; 3vs.1; 1vs.3. The matches (and score) were: Team A vs. team B (1–0); Team A vs. Team C (1–0); Team A vs. Team D (1–0); Team E vs. Team A (2–1); and Team F vs. Team A (0–0).

We computed the relative frequencies of the simplices structures at Level *N* + *1* for players in both teams (Figure 2). The most frequently occurring simplices structures in the 5 matches: 1vs.1; 2vs.1; 1vs.2; 2vs.2; 3vs.1; 1vs.3. These results reveal that the most frequently occurring simplices structures are similar in every match. Around 25% of the simplex structures corresponded to 1vs.1, independently of the type of match (home or away) or its final result. The second most frequently occurring simplices structures were 2vs.1 and 1vs.2 (around 10%), followed by 2vs.2 (around 6%), and finally by 3vs.1 and 1vs.3 (around 3%). Among other simplices structures, we could also often find interactions between the goalkeeper and the goal, as identified in 0vs.1 or 1vs.0 structures (around 11%). However, these simplices structures do not reveal a social interaction (i.e., cooperation or competition) and are therefore not compared to other structures.

By computing the frequencies for the “local dominance tag” compound variable it is possible to investigate for each game the most frequent cooperation and competition interactions sets.

**Figure 3:**
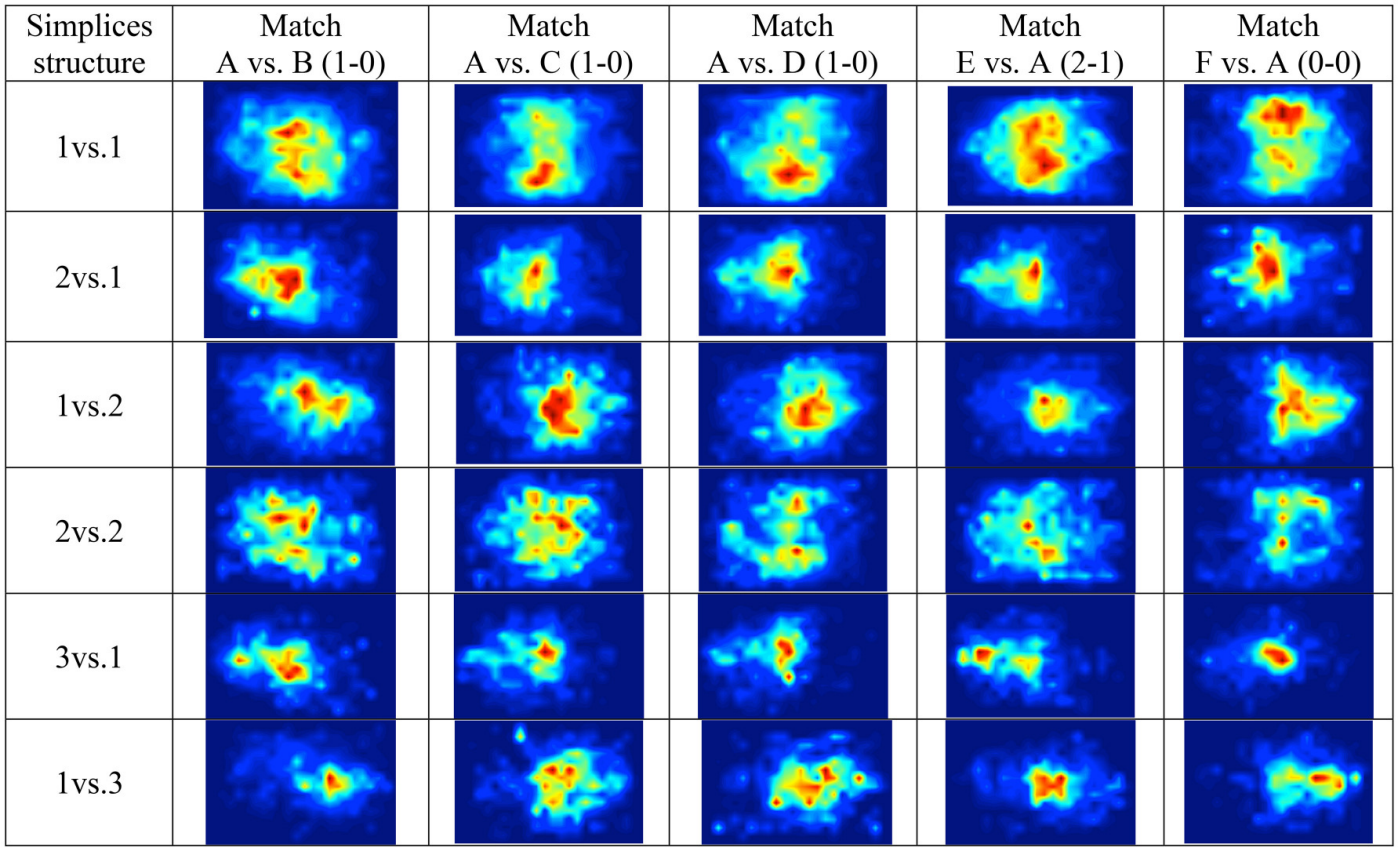
*Heat maps* for field position of the most frequent simplices structures during the matches (when Team A, playing at home, attacks are represented from left to right). The color gradient from red to blue represents the frequency of simplices in that location (from most frequent, red, to not occurring, dark blue).

*Level N* + *1* describes the geographical distribution in the pitch of the most frequently occurring simplices structures, as shown in *heat maps* (Figure 3).

Figure 3 shows that although 1vs.1 is the most frequently occurring simplex tag in every match, the location in the pitch where it can more often be found varies between matches. Simplices, 2vs.1, indicating simultaneous cooperation and competition, occurs mostly in the mid-field, and simplices 1vs.2 occurs mostly in the opponent side of the field.

**Figure 4:**
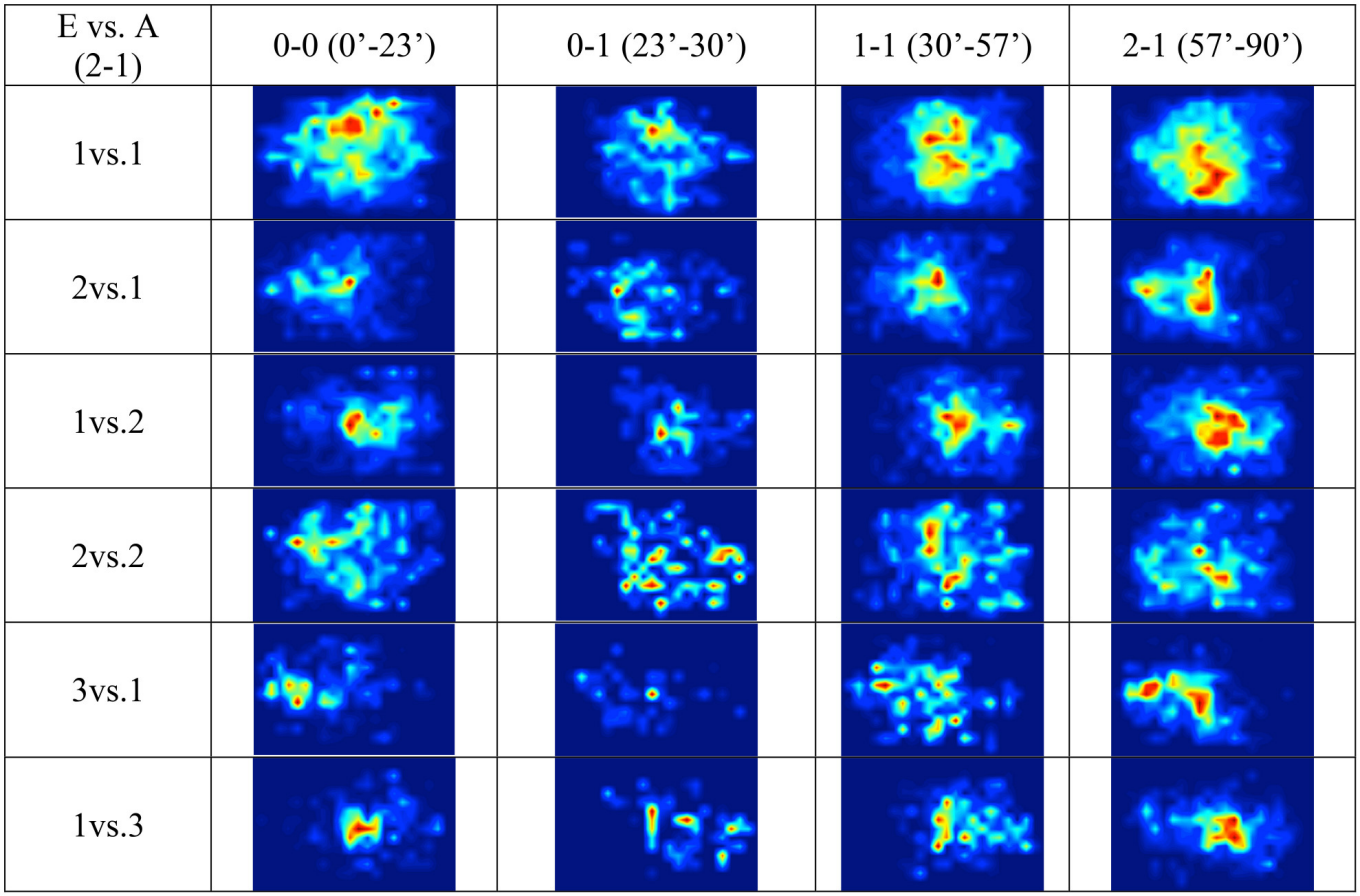
*Heat maps* for the field position of the different simplices structures (visiting team A attacks from the right to the left hand-side). Each column corresponds to a temporal section of the match bound by a score change. The color gradient from red to blue represents the frequency of simplices in that location (from most frequent, red, to not occurring, dark blue).

By identifying the relevant events in a match, such as, changes in the score, at *Level N* + *1* we can capture changes in collective behavior across time. Figure 4 shows the results of this analysis in heat maps corresponding to different sections of the E vs. A match (final result 2–1). For example, these heat maps reveal that the team with the lowest score shows a tendency for a decrease in frequency of 2vs.2 near its own goal. Moreover, the next most frequently occurring simplices, 3vs.1 and 1vs.3, can be found more often close to the goal of the wining team.

**Figure 5:**
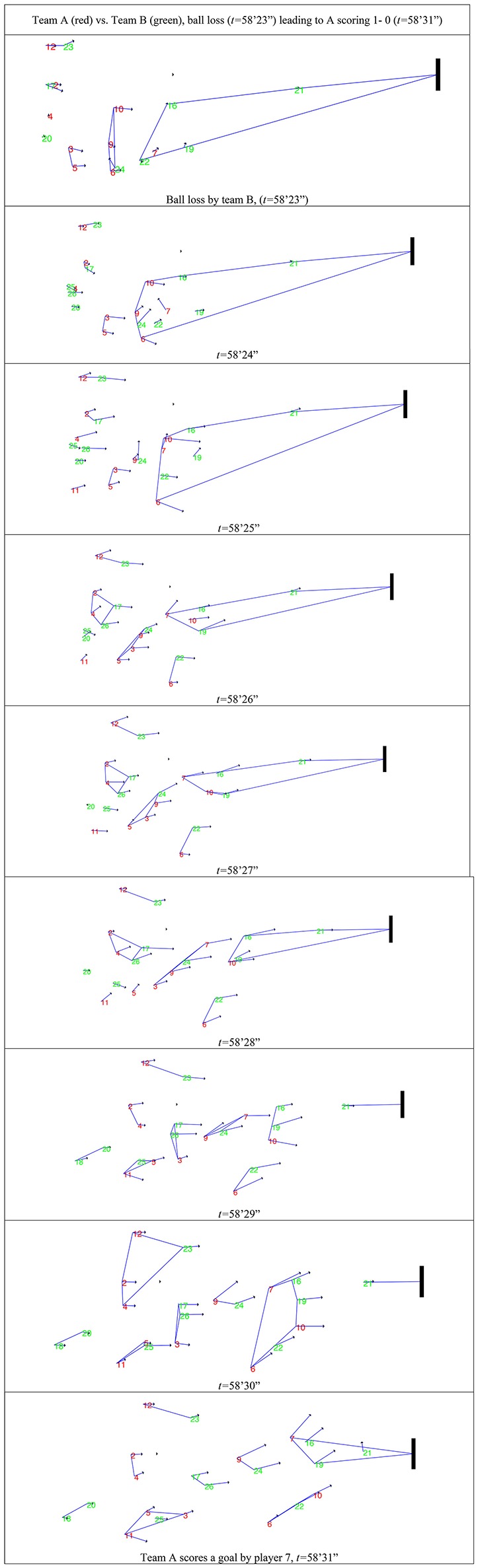
Simplices in a sequence of nine frames (58′23″ to 58′31″) leading to a goal by Team A. Visiting players are attacking from right to left (represented in green), while home players are attacking from left to right (represented in red, including the opponents' goal). A simplex is represented by the polygon (or a line when there are only two players) defining the convex hull (or envelope) that links the nodes (players or goal). A velocity vector for each player is also presented.

*Level N* + *2* captures simplices dynamics, for example, before changes in the score. Here we present an analysis of the simplices having their geographical center closer to the goal. To answer the question “what creates an opportunity for the attackers to score?” simplices reveal how the defenders' local dominance is broken by the attackers. Figure 5 shows an example of local dominance, in which team A (playing at home against B) scores in a counter-attack sub-phase. The play was analyzed in a set of consecutive frames (at 1 Hz) that captured the simplices nearer the goal of interest. A velocity vector computed using consecutive frames was associated to each player to show aggregation or disaggregation, as a player moved toward or away from the simplices geometric center.

The example in Figure 5 shows that, in the frames before a goal is scored, some attacking players (e.g., 6, 7, and 10) increase their speed to place themselves in a better position either to create an invitation for a successful pass or to create a scoring opportunity. On the other hand, defensive players try to maintain or reduce interpersonal distance (e.g., 16, 19, and 22). This is aligned with other studies (Fonseca et al., 2013) where it was observed that attackers tried to increase the interpersonal distance while the defenders tried to reduce it. The consequence of these moves can be captured by simplices' configuration. This is more evident if a player stays in the same simplex or moves to another simplex. Changes in players' velocity leads to break (disaggregate) or maintain (aggregate) the simplex's integrity when they move away or toward the simplex geometric center, respectively.

**Figure 6:**
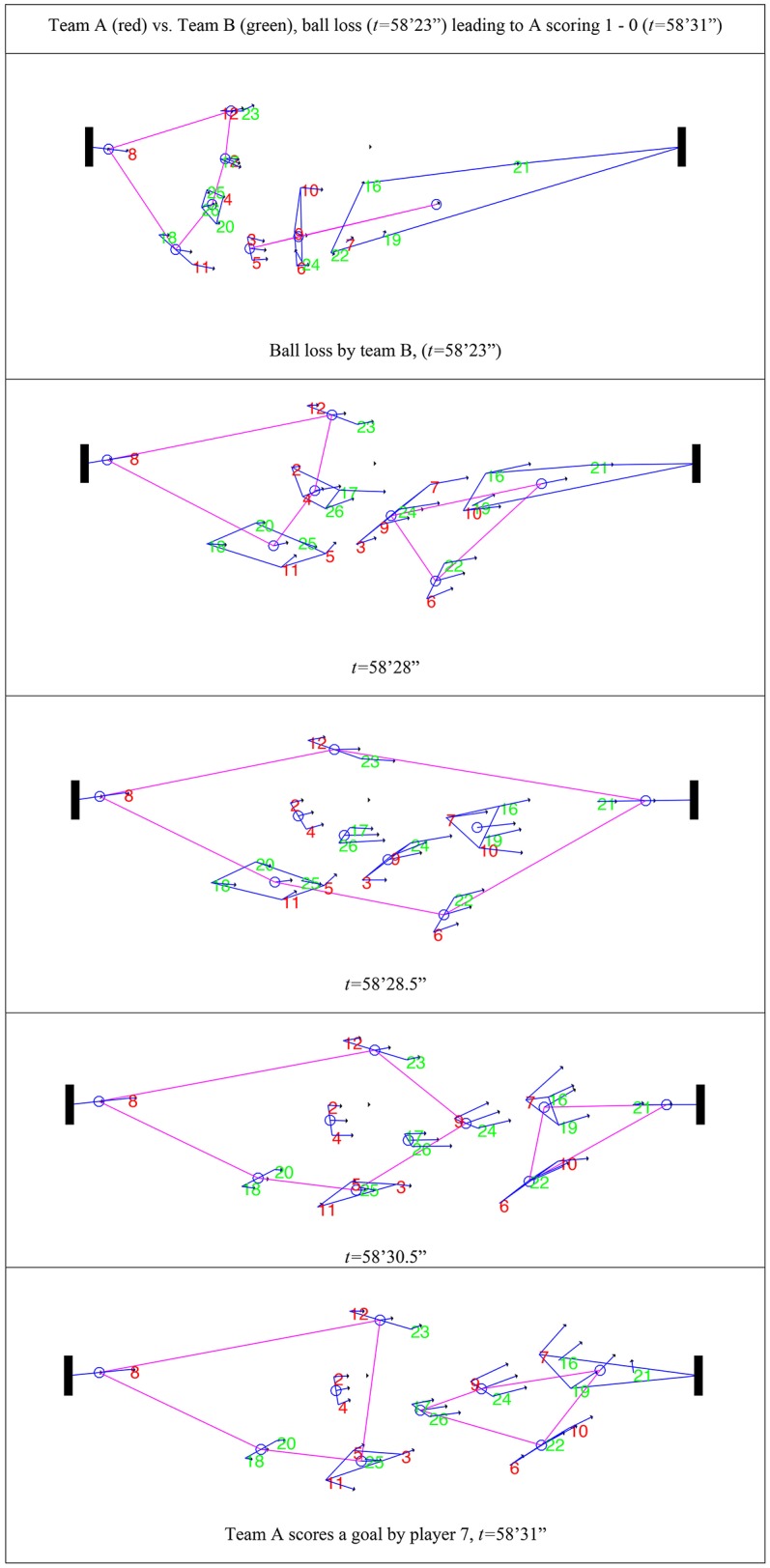
Higher-order simplices (simplices of simplices) in a sequence of five frames before team A scores a goal. Higher-order simplices are represented by the polygon (and lines) forming the convex hull (−) that connects the geographical centers of the *N* + *1* simplices. See Figure 5 legend for the codes for players, their velocity, and simplices.

*Level N* + *3* indicates how simplices interact between them, thereby creating higher-order simplices. These simplices form by aggregation of Level *N* + *1* simplices based on the proximity criterion of their geographical centers (Figure 6). To uncover the changes in simplex structures leading to goal scoring, higher-order simplices (Figure 6, purple polygons) were analyzed for the frames where significant changes occurred in the Level *N* + 3 structures (simplices of simplices).

The example of *Level N* + *3* analysis in Figure 6 also reveals the connections between players before a goal was scored. The simplex formed by the goalkeeper and the goal is connected with other simplices, as the goalkeeper tries to align with the closest simplice while maintaining the link with the goal. Figure 6 also shows how the simplices furthest from the goal are connected with simplices more directly involved in the attacking phase (i.e., closest to the goal). Other information that can be extracted from *Level N* + *3* is how fast changes in the link with the goal can occur, and which simplices are “disconnected,” for example, on one side of the field.

## Discussion

The different levels of analysis of a hypernetwork can capture various degrees of team behavior dynamics, from player, to simplices, and to interactions between simplices across space and time.

At *Level N* + *1*, we could identify the types of simplices occurring more often in a match, independently of their score or context (home or away). The most frequently occurring simplex was 1vs.1, followed by 1vs.0 and 0vs.1. The latter represents the link between the goalkeeper and the goal. Also occurring frequently were simplices with an unbalanced number of players, 2vs.1 and 1vs.2 (~10%), followed by the 2vs.2 simplices (~6%), and finally by the 3vs.1 and 1vs.3 simplices (~3%).

Important interpretations can be inferred from the simplices at *Level N* + *1* when space and time, or contextual variables (home or away match) are considered. For example, team A won three home matches (all with score 1–0) but tied (score 0–0) or lost (score 2–1) in away games. The 1vs.1 simplices tend to occur in the mid-field and on the right of the attacking direction of team A (Figure 3). However, in the match lost against team E, 1vs.1 simplices were more dispersed and toward the left side of the pitch. Another frequently occurring simplex with a balanced number of players was 2vs.2, for both teams (Figure 3). Interestingly, these simplices also had a unique distribution in the match lost against team E, as they occurred more toward the center of the pitch and the opponent middle field. Additionally, these structures differed from match to match, showing the emergent properties of complex adaptive systems, specifically the context dependency (opponents and scoring evolution; Araújo and Davids, 2016).

Concerning simplices with an unbalanced number of players, 2vs.1 occurred more often in the center of the pitch and in the opponent middle field (similarly to 2vs.2 in the match lost against team E). The 1vs.2 simplices were also detected more often in the middle fields. Simplices 3vs.1 were distributed in the center of Team A's middle field, however, in the match against team E, they were more distant from their own goal (in the middle field). In the opposite way, in the matches against teams B and F, there were some notable occurrences of 3vs.1 simplices near team's A goal. Moreover, in these matches, 1vs.3 occurred near the center but more toward team A's middle field, suggesting that team B and F “forced” team A players away from their goal.

The results obtained considered both geographical placement and context dependency, and showed that the use of simplices formation captured match properties, such as, local dominance. These properties emerge in each match event resulting from the local interaction between players of both teams. Multilevel hypernetworks proved to be a useful method in answering to chief problems such as, the relation among micro (e.g., players' positions), meso (e.g., local dominance), and macro levels (e.g., match result). Moreover, the use of hypernetworks allows that the analysis can consider more than the typical (in SNA) *2*-ary relations between players. These contributions fulfill previous gaps in interpersonal coordination research (Passos et al., 2016).

The analysis of the dynamics of simplices interactions at *Level N* + *2* revealed abrupt changes in the speed and direction of player vectors near the goal. These changes showed a tendency to be associated with transformations in simplex structure, for example, when an attacker passed through the defenders to score, or when a player disconnected from one simplex to interact with another (to balance or unbalance the simplex). The example in Figure 5 analyzed a change in the score that resulted from a ball lost by team B in team A's middle field that led to a successful counter attack (with a goal scored). This event was characterized by transformations in the simplices' structure occurring within the short duration of the counter attack (9 s, from 58′23″ to 58′31″). Next we present the set of simplices (σ) and their evolution for these 9 s leading to a goal being scored by Team A (at 58′31″). Simplices containing the player who scored the goal are identified with (S). Simplices containing the goal are identified with (G).

$$\begin{array}{l} \sigma_{1,\;58^{\prime }23^{\prime \prime }}\langle a_{3},\;a_{5}\rangle +\sigma_{2,\;58^{\prime }23^{\prime \prime }}\langle a_{9},\;a_{6},a_{10},d_{24}\rangle \\ \;+\;\sigma_{3,\;58^{\prime }23^{\prime \prime }}\langle a_{7},\;d_{22},d_{16},d_{19},d_{21};(G,S)\rangle \\ \sigma_{1,\;58^{\prime }24^{\prime \prime }}\langle a_{3},\;a_{5}\rangle +\sigma_{2,\;58^{\prime }24^{\prime \prime }}\langle a_{9},\;a_{6},a_{10},d_{24},a_{7},\;d_{22},d_{16},\\ \;d_{19},d_{21};\left(G,S\right)\rangle \\ \sigma_{1,\;58^{\prime }25^{\prime \prime }}\langle a_{3},\;a_{5}\rangle +\sigma_{2,\;58^{\prime }25^{\prime \prime }}\langle a_{9},\;d_{24}\rangle \\ \;+\;\sigma_{3,\;58^{\prime }25^{\prime \prime }}\langle a_{6},a_{10},a_{7},\;d_{22},d_{16},d_{19},d_{21};(G,S)\rangle \\ \sigma_{1,\;58^{\prime }26^{\prime \prime }}\langle a_{3},\;a_{5},a_{9},\;d_{24}\rangle +\sigma_{2,\;58^{\prime }26^{\prime \prime }}\langle a_{6},\;d_{22}\rangle \\ \;+\;\sigma_{3,\;58^{\prime }26^{\prime \prime }}\langle a_{10},a_{7},\;d_{16},d_{19},d_{21};(G,S)\rangle \\ \sigma_{1,\;58^{\prime }27^{\prime \prime }}\langle a_{3},\;a_{5},a_{9},\;d_{24}\rangle +\sigma_{2,\;58^{\prime }27^{\prime \prime }}\langle a_{6},\;d_{22}\rangle \\ \;+\;\sigma_{3,\;58^{\prime }27^{\prime \prime }}\langle a_{10},a_{7},\;d_{16},d_{19},d_{21};(G,S)\rangle \\ \sigma_{1,\;58^{\prime }28^{\prime \prime }}\langle a_{3},\;a_{7},a_{9},\;d_{24};(S)\rangle \;+\sigma_{2,\;58^{\prime }28^{\prime \prime }}\langle a_{6},\;d_{22}\rangle \\ \;+\;\sigma_{3,\;58^{\prime }28^{\prime \prime }}\langle a_{10},\;d_{16},d_{19},d_{21};(G)\rangle \\ \sigma_{1,\;58^{\prime }29^{\prime \prime }}\langle a_{3},\;d_{17},\;d_{26}\rangle +\sigma_{2,\;58^{\prime }29^{\prime \prime }}\langle a_{9},a_{7},\;d_{24};(S)\rangle \\ \;+\;\sigma_{3,\;58^{\prime }29^{\prime \prime }}\langle a_{6},\;d_{22}\rangle +\sigma_{2,\;58^{\prime }29^{\prime \prime }}\langle d_{21};(G)\rangle \\ \sigma_{1,\;58^{\prime }30^{\prime \prime }}\langle a_{3},\;d_{17},\;d_{26}\rangle +\sigma_{2,\;58^{\prime }30^{\prime \prime }}\langle a_{9},\;d_{24}\rangle \\ \;+\;\sigma_{3,\;58^{\prime }30^{\prime \prime }}\langle a_{6},a_{7},a_{10,}d_{16},d_{19},\;d_{22};(S)\rangle +\sigma_{2,\;58^{\prime }30^{\prime \prime }}\langle d_{21};(G)\rangle \\ \sigma_{1,\;58^{\prime }31^{\prime \prime }}\langle a_{9},\;d_{24},\rangle +\sigma_{3,\;58^{\prime }31^{\prime \prime }}\langle a_{6},a_{10,},\;d_{22}\rangle \\ \;+\;\sigma_{2,\;58^{\prime }31^{\prime \prime }}\langle a_{7},d_{16},d_{19},d_{21};(G,S)\rangle \end{array}$$

The results show that certain moves performed by the player who scored the goal (player *a*_7_) had significant impact on some simplices transformations, for example, at instants 58′27″, 58′28″, 58′29″, 58′30″, and goal scored. Player *a*_10_ had an important role in promoting balance in the simplex that scored the goal (with player *a*_7_), by maintaining defender *d*_19_ distant from his teammate *d*_16_. Moreover, player *d*_19_ appeared to be facing the defender's dilemma, hesitating between defending his opponent (player *a*_10_) and supporting his teammate (player *d*_16_). Player *d*_24_ was also essential in the attack play leading to the goal scored, as he lost the ball but kept pursuing it, almost reaching player *a*_7_ and thereby including him into his simplex. Finally, player *a*_6_ broke the central simplex (containing teammate *a*_7_) by attracting a defender toward him and hence reducing the number of players in the central middle field.

Results showed that by considering the temporal sequence of simplices transformations during critical events of the match (e.g., from ball recovery to scoring a goal) the dynamics of interaction among players is captured. Moreover, it is possible to analyze how interactions among players led to changes in simplices' structures and, consequently to such critical events (e.g., a goal scoring opportunity). Multilevel hypernetworks offer a fine temporal grain of analysis of how the micro-meso-macro level relationships emerge.

*Level N* + *3* clarified the dynamics of team behavior by considering the entire set of simplices, including the interactions between them (which form simplices of simplices). This level of analysis revealed the connections of players with simplices during a match. We found that the goal has an “anchoring effect” toward the goalkeeper, however, this simplex also connected with the nearer simplex (0vs.1 represents the home team and 1vs.0 the visiting team). Some simplices seemed to disconnect during critical situations, for example, when other simplices were close to the goal. This may be explained by an intentional reduction in speed by the attacking players to try and maintain the nearest defenders away from teammates (Figure 6).

This study showed that the hypernetworks' analysis by considering simplices of simplices reveal the degree of connection between sub-sets of players.

## Conclusions and limitations

We have applied multilevel hypernetworks analysis, and a set of associated compound variables, to selected soccer matches by using positional variables for all players involved.

The interactions between players, as well as the sets of these interactions (simplices), were assessed based on interpersonal distance, more specifically *spatial proximity* and *instant speed* relational variables. Each player is therefore linked to his closest player (or goal, for the goalkeeper) and at higher levels, simplices are also linked to their closest simplices. The vectors representing the players' speed can represent the emergent moves from the players in order to search for new interactions or escape from others. These two “interaction variables” allowed for a deeper analysis of the structures and coordination levels emerging from the game.

Our results revealed a pattern in these interactions' dynamics that was independent of the type (home or away) and score of the match. Specifically, in every match analyzed the most frequently occurring simplices structures were, by decreasing order of frequency, 1vs.1, 2vs.1 and 1vs.2, 2vs.2, and finally, 3vs.1 and 1vs.3.

However, these simplices show differences in their distribution on the pitch, and this is particularly evident for unbalanced simplices such as, 2vs.1, 1vs.2, 3vs.1, and 1vs.3. These differential distributions are consistent with the match result (wins vs. losses) and the opponent team's strength.

We analyzed the changes in local dominance at *Level N* + *2* associated with critical events (e.g., score changes) and found that dramatic speed changes can be detected in the players of simplices directly linked to the event (goal scored). Velocity is therefore the variable that allows players to improve their positioning to score or to unbalance the situation.

Finally, our last and global analysis level revealed how all the simplices were connected, but most importantly, it enabled to permanently connect all the simplices into larger hypersimplices, including the goal and goalkeeper simplex, and also the defenders and attackers who were distant from the goal.

These results may significantly contribute to improve training and playing strategies. We highlight the importance of mastering 1vs.1 situations (with and without the ball), as this structure occurs more frequently in all types of matches. For example, coaches could design exercises to train players to rapidly transform any structure into a 1vs.1 structure. Unbalanced situations such as, 2vs.1 and 3vs.1 typically reveal which team is dominating the match, particularly when those structures occur on the attacking side of that team's field. Thus, designing training exercises that create an overload for the attacking team may allow players to better adapt to such situations in a match. Finally, we found that as an attacking team moves closer to the goal, changes in player speed become more pronounced. It is therefore likely that encouraging such speed changes during training may facilitate the players' positioning inside finishing areas during a match.

Moreover, when players are connected with other players (in cooperation or competition) forming simplices, where the smaller simplices are also connected with other simplices, team coordination develops due to attunement to shared affordances and the creation of team synergies (Araújo and Davids, 2016). Training sessions may benefit from using the present analysis (e.g., most frequent cooperation/competition tag sets) and consequently design training activities that promote collective learning among groups of players (Travassos et al., 2016).

In the context of this article the criterion, closest player, for the formation of hyperedges was the only one used. The results presented at different levels of analysis are therefore conditioned and limited by this criterion. At the same time all these results where possible with only this parsimonious criterion and without any other assumptions.

Other limitation of the study is that there is no data about ball positioning, nor about “ball flux” (e.g., passes between the players). This type of interactions between players could be included by extending the proposed method with additional layers. In such layers, ball flux could be represented either as a link between players' or simplices, or alternatively as an additional term in the relationship, *R*, of the simplices.

Multilevel hypernetworks is a promising framework for soccer performance analysis that reveals important features of cooperative and competitive interactions during attacking plays. By considering space and time in multilevel analyses involving interactions between two or more players, we can obtain a richer understanding of real-world complex systems.

## Author contributions

JR, main contribution regarding theoretical approach, method and results production. RL, significant contribution regarding method, software computation, and results production. PM, significant contribution on results reading and discussion and the impact to practitioners. DA, significant contribution regarding performance analysis and the general impression.

### Conflict of interest statement

PM is affiliated with the commercial company, City Football Services. The other authors declare that the research was conducted in the absence of any commercial or financial relationships that could be construed as a potential conflict of interest. The reviewers AL and MD declared their shared affiliation with the handling editor, and the handling Editor states that the process nevertheless met the standards of a fair and objective review
